# On the Observation of the Central Dark Line in the Human Tooth Enamel Crystals Through SEM, TEM, STEM, and AFM Microscopes

**DOI:** 10.1155/ijod/9915505

**Published:** 2026-07-02

**Authors:** J. Reyes-Gasga, Marisa Moreno-Rios, A. Rodríguez-Gómez, Etienne Bres

**Affiliations:** ^1^ Institute of Physics, National Autonomous University of Mexico (UNAM), Circuito de la Investigación s/n, Ciudad Universitaria, Coyoacán, Mexico City, 04510, Mexico, unam.mx; ^2^ Postgraduate Studies and Research, Technological Institute of Pachuca, Avenue Felipe Ángeles Km. 84.5 Venta Prieta, Pachuca de Soto, 42083, State of Hidalgo, Mexico; ^3^ Faculté des Sciences et Technologies, UMET, UMR 8207 CNRS, Bâtiment C6, Université de Lille, Villeneuve d’Ascq, 59650, France, univ-lille1.fr

**Keywords:** atomic force microscopy (AFM), central dark line, crystals, electron microscopy, human tooth enamel, scanning electron microscopy (SEM), scanning transmission electron microscopy (STEM), transmission electron microscopy (TEM)

## Abstract

We examine the image contrasts presented at micrometric and nanometric scales of the scanning electron microscope (SEM), transmission electron microscope (TEM), scanning transmission electron microscope (STEM), and atomic force microscope (AFM) in the tapping mode, looking for the observation of the central feature known as “the central dark line” (CDL) in the structure of human tooth enamel crystals. Since our interest is in inorganic material, mainly at the nanometric scale, the enamel samples for SEM and AFM were polished following a metallographic procedure, using progressively finer silicon carbide papers and etching with orthophosphoric acid. For TEM and STEM, the samples were produced by the focused ion beam (FIB) technique. The CDL images were well recorded using TEM and STEM, but not in SEM and AFM images. SEM images revealed the size and morphology of the enamel crystals, while AFM images revealed spherical and band‐like structures. The observation of the CDL using TEM and STEM suggests that it may represent a potential chemical compositional site.

## 1. Introduction

Human tooth enamel primarily consists of inorganic material (95 wt.%), predominantly hydroxyapatite (HAP, Ca_10_(PO_4_)_6_(OH)_2_), along with organic material (3 wt.%) such as proteins and lipids, and water (2 wt.%) [1]. At the micrometric level, enamel is structured into rods or prisms of 3–5 μm in diameter extending from the enamel–dentin junction to the enamel surface. At the nanometric scale, the prisms are composed of aligned HAP‐related elongated crystals with 50 nm width, 100 nm wide, and 200–500 nm long, approximately [2]. The content of water facilitates the diffusion of other elements, such as sodium and magnesium [1, 2].

Another integral component of enamel is the interprismatic region, which surrounds and encapsulates the prisms [3]. The primary distinction between the crystals in the prisms and those in the interprismatic region lies in orientation: within the prisms, crystals are aligned, whereas in the interprismatic region, their arrangement is less ordered. These structural elements combine to create a resilient enamel tissue capable of withstanding chewing forces and resisting damage.

A noticeable feature at the sub‐nanometer level in enamel crystals is the central dark line (CDL) [4, 5]. The spatial distribution of this line suggests it is in fact a plane, as it is observed both perpendicular and parallel to the c‐axis of the enamel crystals along with the (100) planes. However, the term “central dark line” has been retained through time.

Atomic resolution images of the CDL using transmission electron microscope (TEM) and scanning transmission electron microscope (STEM) reveal a continuous atomic lattice throughout the entire crystalline structure, that is there is no observation of defects such as dislocations or stacking faults [4, 5]. Experimentally observed, the CDL has a thickness between 0.2 and 0.8 nm, depending on the axis of observation, and passes through the OH ions [4, 5]. The significance of observing the CDL lies in that it is a vulnerable site within enamel crystals because during acidic dissolution processes, such as the onset of caries, crystals dissolve preferentially at the CDL site. We are convinced that knowing the structure and physicochemical properties of CDL plays an important role in the study and understanding of the caries process in human teeth, a situation that affects billions of people worldwide, and hence its importance for human health. A historical account of the dark line is given in reference [5].

In this study, we used scanning electron microscope (SEM), TEM, STEM, and atomic force microscope (AFM) to examine and compare the contrast of human tooth enamel crystals at micrometric and nanometric scales, mainly in the observation of the CDL. The goal to achieve is to know if the CDL contrast is observable with all these microscopes, mostly with AFM. CDL observations with AFM have been reported, but there are very scarce and some doubts arise as to whether the reported contrast is actually associated with the CDL structure [6, 7].

In SEM, the secondary electron image contains topographical and morphological information of the sample, while the backscattered electron image provides a density contrast, which is an indicator of compositional variation. The exact chemical identity is determined using an energy‐dispersive X‐ray spectroscopy (EDS). In TEM, the image is performed with electrons after passing through a very thin sample (a thickness of, depending on the sample composition, ~100 nm). If one beam is used for imaging, it can be the transmitted beam (bright‐field) or one diffracted (dark‐field). Dark‐field images are useful for observing individual crystals. If the objective aperture is large enough to allow two or more beams, a phase contrast image is formed by the interference of these beams.

Depending on the resolution of the TEM equipment and the number of beams allowed to pass through the aperture, the image is referred to as a “high‐resolution TEM” (HRTEM) image. In STEM, which combines the capabilities of both SEM and TEM, the image is obtained with detectors that record the intensity of the transmitted and diffracted beams, producing a bright‐field image (STEM‐BF) and annular dark‐field images (STEM‐DF), respectively. An annular detector with an internal detection angle of 10–35 mrad is used for the “annular dark field” (ADF) images. Annular detectors with an internal detection angle of 40–95 mrad detect electrons scattered by the nuclei of atoms, so these images are called “high‐angle annular dark field” (HAADF) or “Z contrast” images. TEM, HRTEM, and STEM techniques are very useful in the study of the internal structure and structural defects of the sample.

The AFM images of a sample are obtained through line‐by‐line scanning detecting the deflection profile between the surface of the sample and an extremely sharp tip of the cantilever. In tapping mode (AC mode), the cantilever oscillates near its resonant frequency, causing the tip to oscillate up and down so that it “taps” the surface of the sample. The feedback signal to the controller is the tip oscillation amplitude. Therefore, the AFM microscopy provides information about the morphology, topography, and surface roughness of the observed samples [6, 7].

Therefore, in this work, we sought to observe the contrast of the CDL in human dental enamel crystals at the micrometer and nanometer scales using SEM, TEM, and STEM. TEM techniques included bright‐field (TEM‐BF) and dark‐field (TEM‐DF) imaging, along with high‐resolution transmission electron microscopy (HRTEM). For STEM, we used bright‐field (STEM‐BF) and dark‐field (STEM‐DF) imaging, and for AFM, we used tapping mode.

## 2. Experimental Procedures

Enamel from six permanent human molars of adult patients (older than 25 years old) extracted for orthodontic reasons was used in this work. Institutional Review Board (IRB) approval FMED/CI/SPR/083/2015 for use of human teeth approved by the University of Mexico, in accordance with the Committee on Publication Ethics (COPE) guidelines, includes written informed consent from patients about this type of study.

Teeth were free of caries and white spots. Variables such as variations in dietary history, fluoride exposure, or undetected early subsurface lesions, which could affect the crystalline structure or visibility of the CDL, were not considered. Teeth were checked with the Zeiss light microscope model Axiovert 25 to ensure they were visually free of caries. They were washed three times with distilled water using the Branson ultrasonic equipment model 1510 and stored in distilled water for aftercare.

The procedure for preparing SEM, TEM, STEM, and AFM samples involved transversal (parallel to the tooth surface) and longitudinal (lingual–labial direction) cutting enamel specimens. To facilitate handling, a mixture of NICTONE monomer and methyl methacrylate polymer (Quarz R2V) was prepared, and teeth were immersed in the solution and allowed to dry and harden. Each sample was manipulated to a specific type of analysis without additional selection.

### 2.1. Bulk, SEM and AFM Samples

Teeth were sectioned in rectangular slabs of 5 mm × 5 mm × 1 mm in transversal‐ and parallel‐orientation to the prim‐enamel direction with a water‐cooled Buehler IsoMet 1000 diamond disk cutter. After sectioning, tooth samples were thinned to about 300 µm in thickness using silicon carbide sandpapers of 35, 25, 18, and 5 µm sizes. After each sandpaper, samples were cleaned in an ultrasonic bath with a 1:1 solution of distilled water (15 mL) and isopropanol (15 mL) for 10 min. To achieve a polished mirror finish, 1 to 0.05 µm alumina powders were used and washed in the ultrasonic bath. Throughout this process, progress was continuously monitored using the Zeiss light microscope model Axiovert 25.

After achieving a mirror finish, the samples were etched with 36% orthophosphoric acid for 3 min to reveal their structure. Finally, the resin was removed by heating to 60°C and washed with the 1:1 solution of distilled water (15 mL) and isopropanol (15 mL) for 20 min. For SEM and AFM, the enamel samples were set on the SEM and AFM holders, respectively, with carbon double‐sided tape and coated with a thin gold film in a vacuum evaporator. For AFM, there was no need to coat the sample. In addition to bulk samples, powder enamel samples were produced using dental milling machines.

For SEM observation, a JEOL JSM‐7800F (Jeol, Tokyo, Japan) Schottky field emission scanning electron microscope (FE‐SEM) was used. This microscope offers a resolution of 1.0 nm (at 15 kV), and it can achieve 0.1 nm resolution at a low voltage (<1 kV) in secondary electron imaging mode. For AFM observation, Oxford Asylum MFP‐3D Origin equipment (Asylum Research, Oxford Instruments, California, USA) with an Arrow UHF tip was used in tapping mode in air, dried, and at room temperature. The constant spring force of probes was 40 N/m. The AFM images were obtained with a resonance frequency of 120–190 kHz, a resonance amplitude of 600 mV, a set point of 0.5 V, and scanning speeds of 2.5 Hz with 256 lines per image. Height, amplitude, and phase channels were obtained with image acquisition of 256 × 256 pixels.

### 2.2. TEM and STEM Samples

For TEM and STEM, focused ion beam (FIB) equipment was used to produce lamellas from the mirror‐finished samples in cross‐ and parallel‐orientation to the prim‐enamel direction. The FIB‐FEI QUANTA 200‐3D equipment (Thermo Fisher Scientific, Hillsboro, Oregon, USA) was employed using a field emission electron gun and a gallium ion beam source. During preparation, the area of interest was coated with a platinum layer and the samples were cut using the gallium ion beam while in situ observed with SEM. The acceleration voltage for coarse milling was up to 30 kV for both beams, with a probe current of 200 nA for the electron beam and 5 nA for the ion beam. Afterward, the TEM lamellae were both sides milled with an ion beam at 15 kV and a beam current of 0.5 nA. For in situ manipulation of TEM samples, the OmniProbe 100.7 (Oxford Instruments, California, USA) micromanipulator equipped with gas injection systems for platinum deposition was utilized.

For TEM and STEM observations, a JEOL 2010‐FasTEM microscope (Jeol, Tokyo, Japan) with a field emission electron gun, a resolution of 0.2 nm operated and at 200 kV was used. Additionally, a Titan‐300 FEI microscope (Thermo Fisher Scientific, Hillsboro, Oregon, USA), equipped with a field emission filament and operated at 300 kV, was also used.

## 3. Results

### 3.1. Transversal‐Orientation and Powder Samples

**Figure 1:**
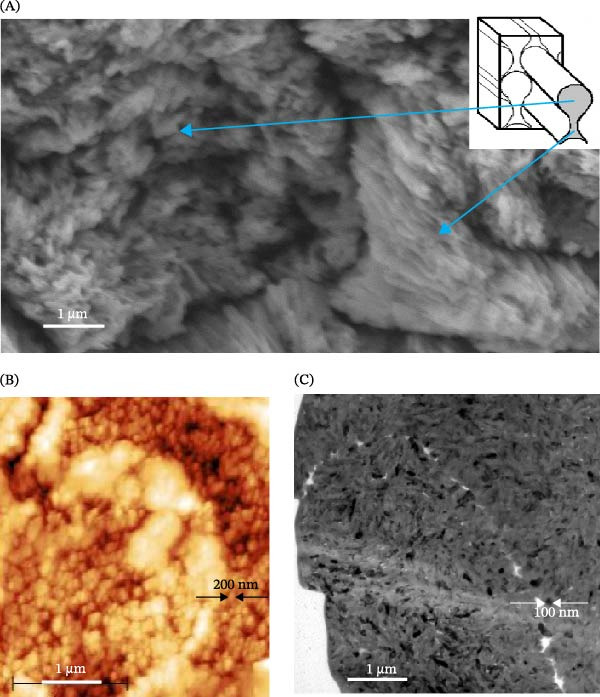
SEM (A), AFM (B), and TEM (C) images of the “head” of a human tooth enamel prism in transversal‐direction samples. The inset in (A) shows a schematic drawing illustrating the prisms arrangement in enamel and the “head” and “basis” of the prism.

The “head” and “basis” of the human tooth enamel prisms are easily observed using SEM, TEM, STEM, and AFM. Figure 1 shows a transversal‐sectional view (transverse direction sample) at the micron scale of the prism’s head. SEM and AFM images depict a rough surface morphology, while the TEM image reveals the nanometric crystals that constitute the enamel prism. It is noteworthy that the AFM images show the enamel crystals as pseudospheres. The roughness measured by AFM ranged between 8 and 39 nm.

**Figure 2:**
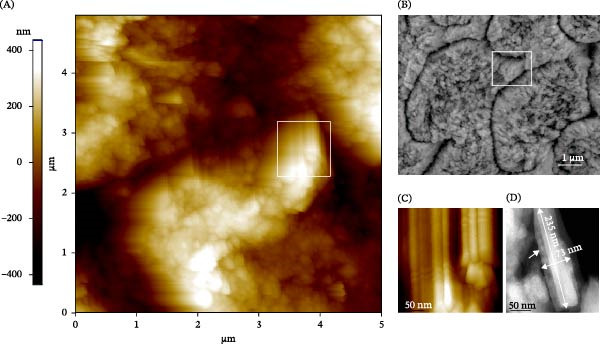
AFM (A) and SEM (B) images of human tooth enamel prisms in a transversal‐direction sample. The square in (A) and (B) indicates the group of periphery crystals shown in detail in (C). (D) SEM image of a periphery nanocrystal in a powder sample. Note the pseudo‐spherical shape at the tip (indicated by the arrow in C) and the ~20 nm spheres on the side of the crystal (indicated by the arrow in D).

Two types of nanocrystals were observed in the enamel prisms: one is located on the periphery of the prism and the other inside the prism. Figure 2 shows these crystals in SEM and AFM images. The squared area in the AFM image and the SEM image (Figure 2A,B) identifies the group of periphery crystals shown in detail in the AFM image (Figure 2C). Figure 2D shows the SEM image of a periphery crystal observed in a powder sample. Additionally, spheres of ~20 nm are visible on the sides of the crystal of Figure 2D.

The bands or domains observed in the AFM image of Figure 2C resemble those reported by Kirkham et al. [8] and Robinson et al. [9]. In fact, these bands are not perpendicular to the long axis of the crystals but make an angle producing a spiral‐like contrast. These bands are observed only in AFM images, and we made sure that they are not an artifact produced during the acquisition of the AFM images since they were observed in different crystals and always in a spiral shape, not horizontal to the crystals.

**Figure 3:**
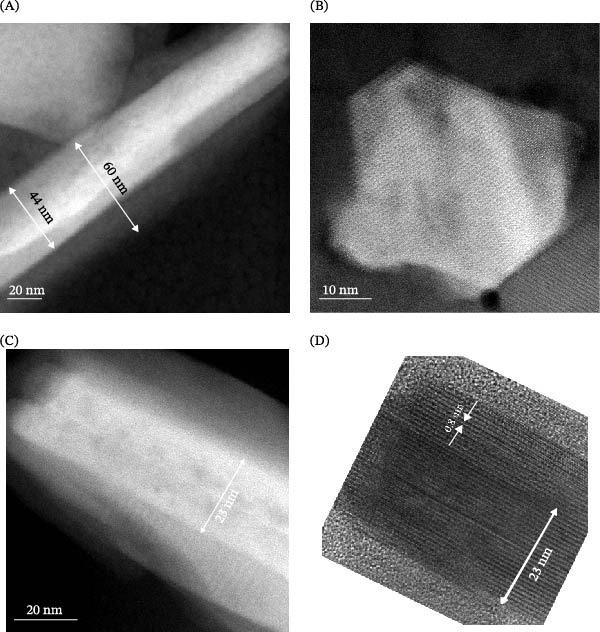
STEM‐DF (A–C) and STEM‐BF (D) images of the periphery nanocrystals of the human tooth enamel prism. (A) Longitudinal view, (B) transverse view. Note the white contrast of the CDL in (C) and the black contrast in (D). In (D) the CDL is observed together with atomic lines with a periodicity of 0.8 nm corresponding to the (100) planes of the hydroxyapatite unit cell.

Figure 3 shows the dark field (STEM‐ADF) and the bright field (STEM‐BF) STEM images of the periphery crystals of the enamel prism observed in the powder sample. The transverse direction image (Figure 3B) reveals a pseudo‐trapezoid transversal shape of these crystals. Note in Figure 3C,D the contrast of the CDL. The lines parallel to the CDL have a periodicity of 0.8 nm, corresponding to the (100) atomic planes of the hydroxyapatite unit cell.

**Figure 4:**
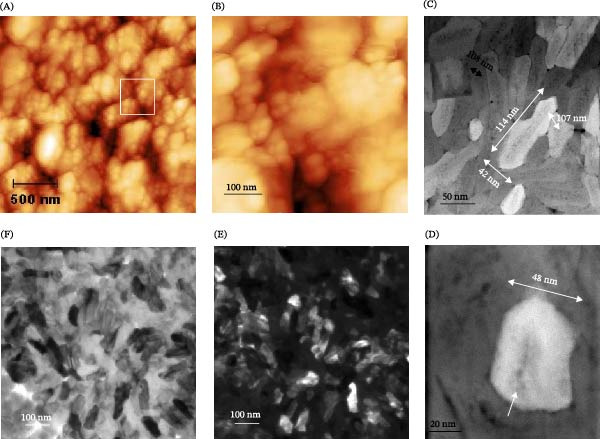
AFM (A, B), STEM‐DF (C, D) and bright field and dark field TEM (E, F) images of the nanocrystals inside the human dental prism in transverse direction samples. The rectangle shown in (A) is magnified in (B). The CDL is visible in (C) and (D).

Figure 4 shows the AFM (Figure 4A,B), the STEM‐DF (Figure 4C,D), and the bright‐ and dark‐field TEM (Figure 4E,F) images at the nanometric scale of the crystals inside the human dental prism in transverse direction samples. In AFM images, these nanocrystals appear as pseudo‐spheres, while they are observed as individual crystals in TEM and STEM images. The CDL is visible in the crystals in STEM images. Also note the “core–shell‐type” contrast around the CDL exhibited in the STEM images.

**Figure 5:**
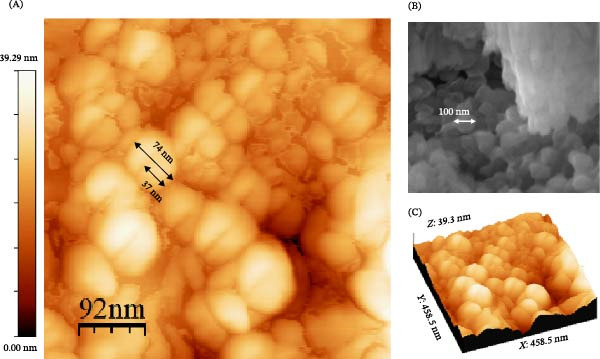
AFM (A) and SEM (B) images of the nanocrystals inside the human enamel prism. (C) 3D image from (A).

When observing with SEM and AFM at higher magnification (reducing the scanned area), the nanocrystals inside the enamel prism appear as pseudo‐spherical in shape, resembling “chickpeas.” Figure 5 shows both the AFM (Figure 5A) and SEM (Figure 5B) images of these crystals. In the case of AFM, there is a line that crosses the central part of the chickpeas‐like crystals. However, this line is presented in the same orientation and always dividing the crystals halfway, which raises doubts about whether it is or not the CDL, since the CDL is always randomly oriented, as observed in Figure 4C. In TEM, the CDL is always observed. However, a more detailed analysis of the line observed in Figure 5A is required to determine its origin and whether or not it is related to the CDL, whether it is a scanning artifact in the AFM or a polishing defect.

### 3.2. Parallel‐Orientation Samples

**Figure 6:**
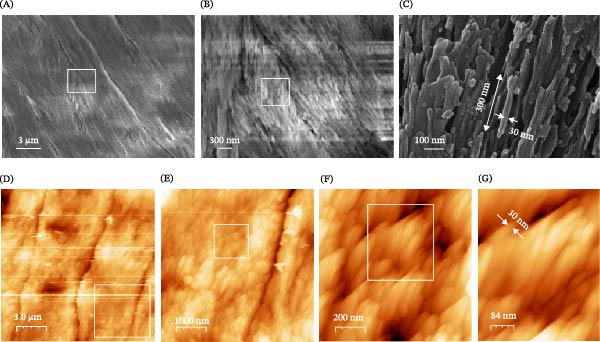
SEM (A–C) and AFM (D–G) images of human enamel prisms in parallel‐to‐prism direction samples at different magnifications. The square in each image indicates the area amplified in the following image.

In parallel‐orientation direction samples, enamel prisms and crystals appear elongated. Figure 6 shows the SEM and AFM images of enamel prisms in this direction at micrometric and nanometric scales. Both SEM and AFM images reveal individual enamel nanocrystals, but none of them display the CDL.

**Figure 7:**
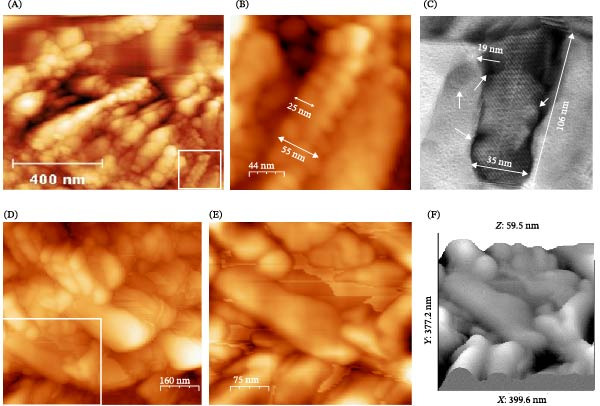
AFM (A, B, D, E, and F) and TEM (C) images of human tooth enamel nanocrystals in parallel‐to‐prism direction samples. The square in (A) indicates the zone shown in (B). (D–F) AFM images of enamel crystals at higher magnifications. The square in (D) indicates the amplified area shown in (E). (F) 3D image from (E). Note in (C) the pseudo‐spherical structures around the nanocrystal (indicated by arrows) and the presence of the CDL.

In some AFM images (as shown in Figure 7A,B), enamel nanocrystals were observed surrounded by pseudo‐spherical structures resembling those reported by Robinson et al. [10], Moradian‐Oldak et al. [11], Fincham et al. [12], and Kirkham et al. [8] and related with the amelogenin protein. According to Wang et al. [3], amelogenin self‐assembles via protein–protein interactions can form nanospheres, of ~20 nm in diameter, which stabilize the matrix containing initial enamel crystals and restrict their growth in kinetically preferred directions.

In addition to the CDL, some TEM images also show pseudo‐spherical dark areas (indicated by the arrows in Figure 7C). Considering the contrast formation in TEM (electron diffraction contrast or phase contrast), it is probable that these areas have greater thickness. The presence of pseudo‐spheres may produce a bump‐like surface of the enamel crystals.

**Figure 8:**
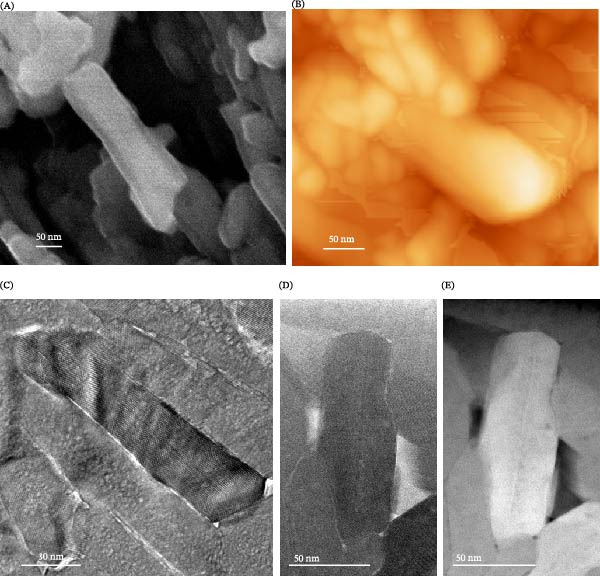
SEM in secondary electron mode (A), AFM in tapping mode (B), TEM in bright field mode (C), STEM‐BF mode (D), and STEM‐DF mode (E) images of human tooth enamel nanocrystals.

Figure 7D–F shows AFM images of human tooth enamel nanocrystals at different magnifications. Compared with the enamel crystal shown in Figure 7C, it is evident that SEM and AFM lack resolution to observe the CDL, or the CDL does not have morphological information to be resolved on the crystal surface. As a summary, Figure 8 shows the SEM, AFM, TEM, and STEM images of almost individual human tooth enamel nanocrystals. Note once again that the CDL is only visible in the TEM and STEM images.

### 3.3. An Alternative Way to Observe the CDL by SEM and AFM

**Figure 9:**
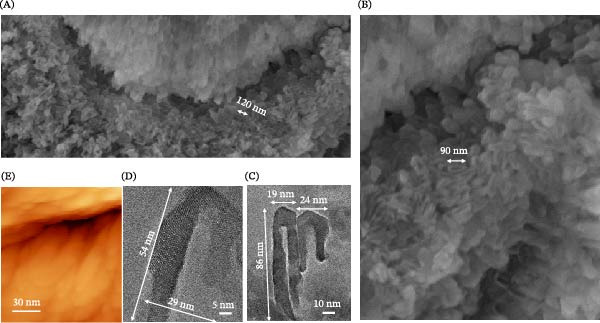
SEM (A,B), TEM (C,D), and AFM (E) images of tooth enamel nanocrystals at different stages of acidic dissolution.

One characteristic of the CDL is its well‐known role in the dissolution of enamel nanocrystals through carious processes, acid exposure, and other substances [6], resulting in a “donut‐like” shape [4, 5]. Figure 9 shows the SEM, TEM, and AFM images of enamel nanocrystals at different stages of dissolution, induced in this case by orthophosphoric acid during sample preparation. Because the dissolution of enamel crystals starts at the CDL [4, 5], this figure suggests that the dissolution process of enamel crystals can be effectively used to study some structural properties of the CDL using AFM and SEM, and the study of partially dissolved enamel crystals using AFM would greatly contribute to understanding the properties of the CDL.

In the transverse direction and after exposure to a pH of 5, Robinson et al. [9] reported that dissolution occurs in the center of enamel nanocrystals along the CDL. Their AFM cross‐sectional images revealed clusters of pseudospheres with a hole in the center. In longitudinally sectioned crystals, they also showed that the extension of the central hole or groove gives them the appearance that the crystals have half the normal diameter, with the two faces appearing as halves of the same crystal, such as shown in Figure 9.

## 4. Discussion

CDL was observed in TEM, HRTEM, and STEM images, but not in SEM and AFM images; that is in “transmited” samples, not in “bulk” samples. This may be due to that the CDL contrast arises from a phenomenon such as phase contrast as those observed in TEM or in STEM, which lack a corresponding surface morphological or topographic feature detectable by AFM and SEM.

The lack of contrast of the CDL in SEM and AFM images suggests either that it is below the resolution of these devices or that its origin could be more chemical than structural. The resolution of the microscopes, of course, plays a crucial role in observing the CDL [4, 5]. The experimental observed thickness for the CDL in HRTEM of corrected aberration has been reported to be between 0.3 and 0.8 nm, depending on the zone axis of observation [4]; however, it is observed between 1 and –2 nm in thickness in the TEM and STEM images of Figures 3, 7, and 8.

In SEM, TEM, HRTEM, and STEM, resolution depends on the electron wavelength, which is related to the acceleration voltage of the microscopes, as well as on spherical aberration. Modern HRTEM microscopes achieve resolutions of around 0.12 nm, while for STEM the reported resolution is around 50 pm. In SEM, the reported resolution for a cold FEG SEM is around 0.1 nm at low voltage, and the quality of the conductive coating applied to the sample is crucial to revealing material details at higher magnifications and resolutions; however, this coating might obscure the original features, especially in biological samples such as tooth enamel. In contrast, for AFM, resolution depends on the size of the probe tip and the equipment stability, making a nanometric‐sized tip essential for this analysis [13]. Therefore, obtaining high‐quality AFM images requires probes with tip radii in the single‐digit nanometer range and slow image acquisition speeds.

Regarding the chemical composition, some hypotheses suggest that the CDL could be a remnant of calcified organic matrix trapped at the center after crystal growth, which also produce stress around it [4], and indicating its presence from the initial stages of mineralization [14]. In STEM‐ADF images, the region surrounding the CDL in crystals appears darker (Figures 4 and 8), creating a “core–shell” type contrast [15]. Maybe the CDL originates from the crystal maturation pathway. Another possibility is that it is adaptive because it accommodates prestress in enamel crystals.

The analysis of atom probe tomography (APT) results have suggested that the center of the enamel crystal, at the CDL site, is a Mg^2+^‐rich intergranular phase and Mg‐ACP phase at grain boundaries between enamel crystals [14, 16–19]. Furthermore, the center of enamel crystals also contains sodium, fluorine, and high concentrations of carbon, likely from carbonate (CO_3_^2−^) [14], although fluorine and carbon are more concentrated around the crystals and not inside them [17–19]. The distinct chemical composition of the CDL compared to the rest of the crystal producing the “core‐shell” type structure suggests the presence of stress fields. All this indicates that it is not possible to observe CDL with SEM and AFM, or that the resolution of these microscopes needs to be significantly improved.

The results also suggest that an alternative way to observe the presence of CDL using SEM and AFM is in partially dissolved enamel crystals after acid treatment. Thus, partially dissolved crystals (such as those seen in Figure 9) provide an alternative method, since the dissolution process modifies the surface morphology. However, it is important to note that the central groove or hole produced in the partially dissolved crystals can also be caused by the carious etching process, thus preventing the image from accurately reflecting the original, undissolved CDL structure.

Further studies using AFM, SEM, TEM, and STEM to analyze the dissolution process could provide additional insights into the structure and functional role of CDL in human tooth enamel. To confirm that the origin of the CDL is a chemical variation, more experiments are needed, or at least references that present APT or EELS data.

## 5. Conclusions

The CDL contrast was observed exclusively in TEM and STEM images but not in SEM and AFM images. This result could be due to two factors: the thickness of the CDL is beyond the resolution of SEM and AFM microscopes, or the CDL only represents a chemically compositional site. The latter could be the case but reports on the observation of the CDL structure indicate that there are structural defects around the CDL such as stress fields, which could be recorded with SEM and AFM microscopes. In particular, the CDL contrast in STEM displays a “core–shell” structure, suggesting both a compositional and structural defect. To elucidate this dilemma, it is necessary to improve the resolution of AFM and SEM microscopes to definitively rule out the non‐observation of CDL contrast by these techniques.

## Author Contributions

Conceptualization: J. Reyes‐Gasga, Marisa Moreno‐Rios, A. Rodríguez‐Gómez, and Etienne Bres. Data curation: J. Reyes‐Gasga. Funding acquisition: J. Reyes‐Gasga. Investigation: J. Reyes‐Gasga, Marisa Moreno‐Rios, A. Rodríguez‐Gómez, and Etienne Bres. Methodology: J. Reyes‐Gasga and Marisa Moreno‐Rios. Project administration: J. Reyes‐Gasga. Supervision: J. Reyes‐Gasga, A. Rodríguez‐Gómez, and Etienne Bres. Writing – original draft: J. Reyes‐Gasga, Marisa Moreno‐Rios, A. Rodríguez‐Gómez, and Etienne Bres. Writing – review and editing: J. Reyes‐Gasga, Marisa Moreno‐Rios, A. Rodríguez‐Gómez, and Etienne Bres.

## Funding

This study is supported by the Dirección General de Asuntos del Personal Académico, Universidad Nacional Autónoma de México (Grant IN‐100923).

## Disclosure

All authors have read and approved the final version of the manuscript. J. Reyes‐Gasga had full access to all of the data in this study and takes complete responsibility for the integrity of the data and the accuracy of the data analysis.

## Conflicts of Interest

The authors declare no conflicts of interest.

## Data Availability

The authors confirm that the data support the findings of this study are available within the article.
